# Wastewater and environmental sampling holds potential for antimicrobial resistance surveillance in food-producing animals - a pilot study in South African abattoirs

**DOI:** 10.3389/fvets.2024.1444957

**Published:** 2024-10-03

**Authors:** Viivi Heljanko, Musafiri Karama, Amanda Kymäläinen, Paula Kurittu, Venla Johansson, Ananda Tiwari, Matteo Nyirenda, Mogaugedi Malahlela, Annamari Heikinheimo

**Affiliations:** ^1^Faculty of Veterinary Medicine, Department of Food Hygiene and Environmental Health, University of Helsinki, Helsinki, Finland; ^2^Veterinary Public Health Section, Faculty of Veterinary Science, Department of Paraclinical Sciences, University of Pretoria, Pretoria, South Africa; ^3^Centre for Animal Health Studies, Faculty of Natural and Agricultural Sciences, North-West University, Mahikeng, South Africa; ^4^Finnish Food Authority, Seinäjoki, Finland

**Keywords:** antimicrobial resistance, AMR surveillance, wastewater surveillance, food-producing animals, ESBL-producing enterobacterales

## Abstract

Antimicrobial resistance (AMR) poses a significant global One Health challenge that causes increased mortality and a high financial burden. Animal production contributes to AMR, as more than half of antimicrobials are used in food-producing animals globally. There is a growing body of literature on AMR in food-producing animals in African countries, but the surveillance practices across countries vary considerably. This pilot study aims to explore the potential of wastewater and environmental surveillance (WES) of AMR and its extension to the veterinary field. Floor drainage swab (*n* = 18, 3/abattoir) and wastewater (*n* = 16, 2-3/abattoir) samples were collected from six South African abattoirs that handle various animal species, including cattle, sheep, pig, and poultry. The samples were tested for Extended-Spectrum Beta-Lactamase (ESBL) and Carbapenemase-producing Enterobacterales, Methicillin-Resistant *Staphylococcus aureus* (MRSA), Vancomycin-resistant *Enterococci* (VRE), and *Candida auris* by using selective culturing and MALDI-TOF MS identification. The phenotype of all presumptive ESBL-producing *Escherichia coli* (*n* = 60) and *Klebsiella pneumoniae* (*n* = 24) isolates was confirmed with a disk diffusion test, and a subset (15 and 6 isolates, respectively), were further characterized by whole-genome sequencing. In total, 314 isolates (0–12 isolates/sample) withstood MALDI-TOF MS, from which 37 species were identified, *E. coli* and *K. pneumoniae* among the most abundant. Most *E. coli* (*n* = 48/60; 80%) and all *K. pneumoniae* isolates were recovered from the floor drainage samples, while 21 presumptive carbapenem-resistant *Acinetobacter* spp. isolates were isolated equally from floor drainage and wastewater samples. MRSA, VRE, or *C. auris* were not found. All characterized *E. coli* and *K. pneumoniae* isolates represented ESBL-phenotype. Genomic analyses revealed multiple sequence types (ST) of *E. coli* (*n* = 10) and *K. pneumoniae* (*n* = 5), including STs associated with food-producing animals globally, such as *E. coli* ST48 and ST10 and *K. pneumoniae* ST101. Common beta-lactamases linked to food-producing animals, such as *bla*_CTX-M-55_ and *bla*_CTX-M-15_, were detected. The presence of food-production-animal-associated ESBL-gene-carrying *E. coli* and *K. pneumoniae* in an abattoir environment and wastewater indicates the potential of WES in the surveillance of AMR in food-producing animals. Furthermore, the results of this pilot study encourage studying the topic further with refined methodologies.

## 1 Introduction

Antimicrobial resistance (AMR) is a significant global health concern, threatening humans and animals. Multidrug-resistant bacteria can cause hard-to-treat infections and increased mortality due to limited treatment options (1). Furthermore, multidrug-resistant bacteria have been associated with a substantial economic burden because of high healthcare costs caused by prolonged hospital stays, usage of expensive last-resort antimicrobials, and resource-consuming screening and infection prevention measures (2, 3). In recent years, the impact of AMR has become more pronounced, with an estimated 4.95 million deaths attributed to AMR in 2019 worldwide (4). The global burden of AMR is not evenly distributed; it will be particularly high in Africa, where the amount of annual AMR-attributable deaths is estimated to rise to 4.15 million by 2050 (4, 5).

The overuse and misuse of antimicrobials are the main drivers of AMR (1). Notably, the intensive use of antimicrobials in food-producing animals is of particular concern, as more than half of all antimicrobials globally are used in this sector (6). Furthermore, it is common to compensate for poor sanitation or lack of infection prevention and control measures by using antimicrobials (6). In many African countries, antimicrobial usage in food-producing animals is high, especially considering tetracyclines, aminoglycosides, and penicillin (7). Antimicrobials are used for clinical treatment, prophylaxis, and, in some countries, also for growth promotion. Furthermore, a significant proportion of antimicrobials are administered to food-producing animals by animal owners themselves, without proper veterinary supervision and control of the usage (7, 8).

The global emergence of AMR bacteria in animals, such as Extended-spectrum beta-lactamases (ESBL) carrying Enterobacterales, has been evident for years (9). The prevalence of multi-drug resistant *E. coli* in food-producing animals in South Africa is estimated to be high, but only a limited number of studies are available (7). While several African countries have taken steps to develop a national action plan for AMR (10), and research focusing on AMR in food-producing animals in African countries has become more commonplace, surveillance systems vary considerably between countries and remain scanty (7).

Implementation of AMR surveillance in the veterinary field – also in low-and middle-income countries (LMICs) – would provide important information regarding the global trends of AMR, guide the treatment choices, and help proactive intervention and management of AMR. The AMR surveillance system in food-producing animals in the European Union (EU) is well-established and based on caecal samples collected in abattoirs (11). The geographical distribution of animals across the country encourages the centralization of surveillance efforts in abattoirs instead of farms. This reduces logistical challenges and enables the collection of an adequate sample size. Even when focusing the sampling process on abattoirs, obtaining samples from a large population remains laborious and resource-intensive. Wastewater-based surveillance offers a promising and resource-efficient approach for population-level surveillance of AMR (12). Several studies have described antibiotic resistant bacteria in abattoir wastewater. However, the studies have focused on singular abattoirs, and there has been little to no remark on the surveillance potential of the samples as the focus has remained on the potential contamination risks that animal-origin wastewater can cause for the environment (13–15). Thus, wastewater as a population surveillance tool has been utilized mainly in human populations. Nevertheless, wastewater surveillance could offer a rapid and effective method for surveillance of AMR in food-producing animals by enabling the collection of samples without intervention to individual animals or carcasses and with minimal disturbance to the production line.

In this pilot study, we use a combination of selective culturing and molecular methods to study AMR microbes in floor drainages and wastewater of six South African abattoirs. We describe relevant AMR clones and genes in the abattoir environment and wastewater, known to be circulating in food-producing animals. Animals, particularly food-producing animals, play a significant part in the field of AMR, and new perspectives could help to improve global AMR surveillance in the veterinary sector. One of the main objectives of this study is to stimulate the discussion and increase research interest in wastewater and environmental surveillance (WES) of AMR and its extension to the veterinary field.

## 2 Materials and methods

### 2.1 Abattoirs

**Figure 1:**
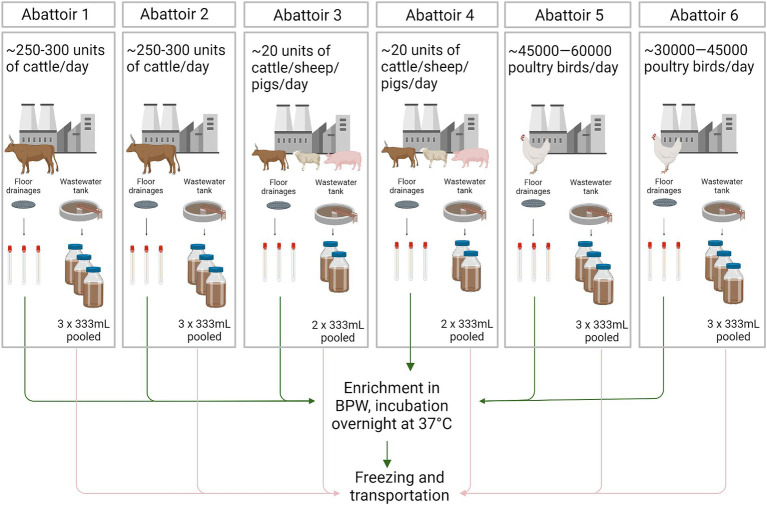
Illustrations of the sample collection and pre-handling of the samples. Arrows describing the workflow for floor drainage swab samples (green) and wastewater samples (pink). BPW, Buffered Peptone water. Figure created with BioRender.com.

Six abattoirs located in the North West province of South Africa were included in the study. Two were cattle abattoirs (abattoirs 1 and 2), two were mixed species abattoirs (cattle, sheep, pigs; abattoirs 3 and 4), and two were poultry abattoirs (abattoirs 5 and 6) (Figure 1). All abattoirs source their animals from their own feedlots. Abattoir 1 also receives animals from farmers throughout the country, and abattoirs 3–6 from farmers across the North West Province of South Africa.

### 2.2 Sample collection, pre-handling, and shipping of the samples

Samples from the floor drainages (swabs, *n* = 18) and wastewater samples (*n* = 16) from the wastewater tanks were collected from each abattoir (Figure 1). All samples were obtained during one sampling day as follows.

In total, three swab (M40 Transystem Amies Agar Gel, Copan Diagnostics, Brescia, Italy) samples from three separate floor drainages and two to three grab samples of wastewater from the wastewater collection tank (333 mL each) were obtained from each abattoir during the sampling day. Samples were transported on ice to the Veterinary Public Health Laboratory, Faculty of Veterinary Science, University of Pretoria, Onderstepoort, South Africa. At the laboratory, swabs were aseptically enriched by incubation in 10 mL of Buffered Peptone Water (BPW) (Oxoid, Basingstoke, Hampshire, United Kingdom) at 37°C for 18–24 h. After incubation, the enriched samples were frozen at-70°C. Wastewater samples were pooled together at the laboratory and frozen at-70°C (Figure 1).

Frozen samples were packed on dry ice and air transported to the laboratory of the Food Hygiene and Environmental Health Department, Faculty of Veterinary Medicine, University of Helsinki, Finland, by a courier company (FedEx™). Samples were unpacked upon arrival and processed further within the same day.

### 2.3 Enrichment and culturing

The floor drainage samples were re-enriched by adding 1 mL of the sample into 9 mL of fresh BPW (Oxoid, Basingstoke, Hampshire, United Kingdom) and incubated for 18–24 h at 37°C. Wastewater samples were enriched by following the same protocol.

Five selective chromogenic agars were used for culturing of the samples: CHROMagar Orientation + ESBL-supplement (referred to as CHROMagar ESBL), CHROMagar mSuperCARBA, CHROMagar VRE, CHROMagar MRSA, and CHROMagar Candida Plus (CHROMagar, Paris, France). Bacteria belonging to ESKAPE-E (16) including, *Enterococcus faecium,* Methicillin-resistant *Staphylococcus aureus* (MRSA), *Klebsiella pneumoniae*, *Acinetobacter baumannii*, *Pseudomonas aeruginosa*, *Enterobacter* spp., and *Escherichia coli*, along with clinically relevant fungal pathogen *Candida auris*, were targeted from the selective agar plates.

**Figure 2:**
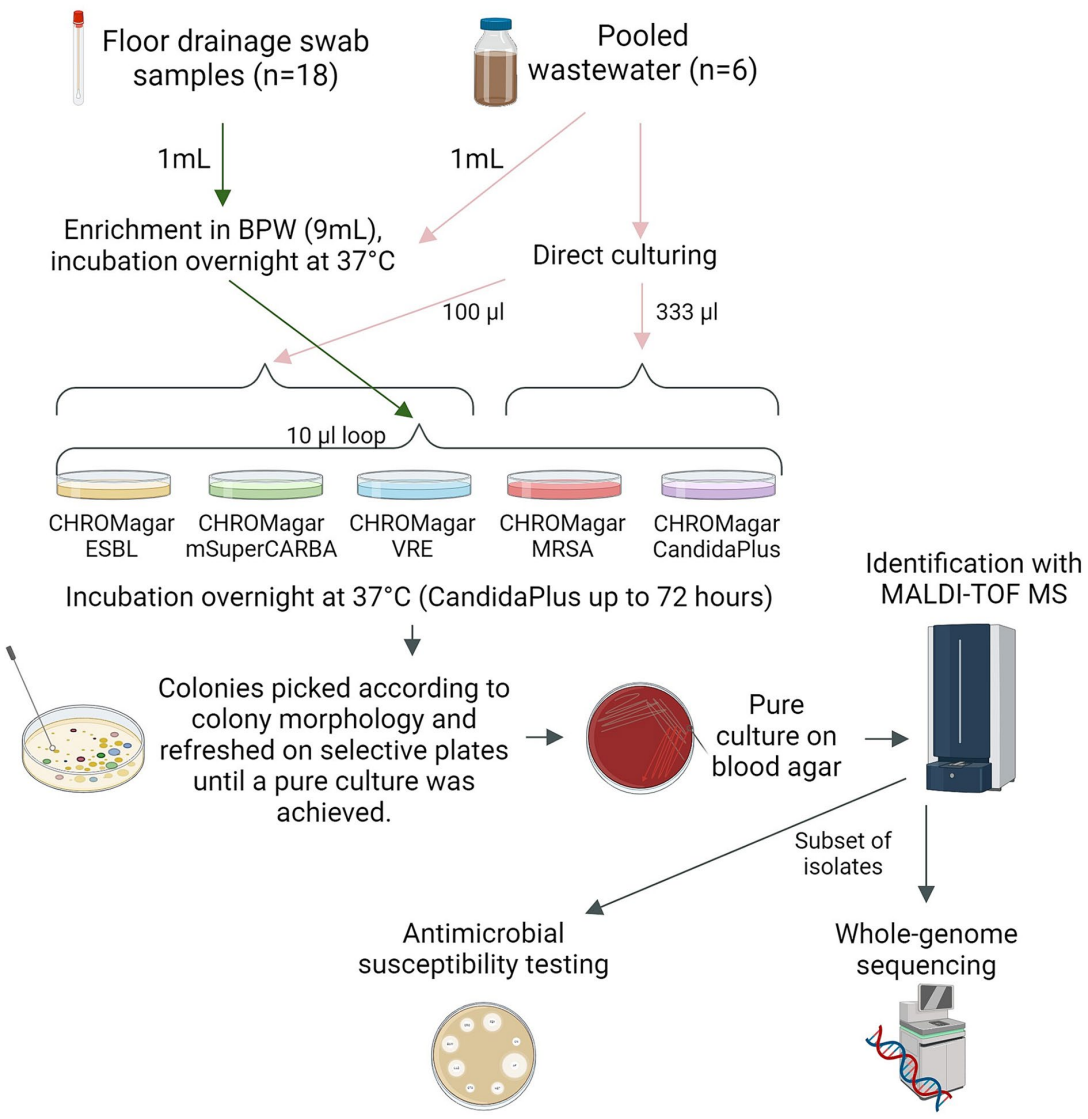
Illustrations of methodology used for bacterial isolation, identification, and characterization. Arrows describing the workflow for floor drainage swab samples (green), wastewater samples (pink), and bacterial isolates (gray). BPW, Buffered Peptone water. CHROMagar ESBL, CHROMagar Orientation + ESBL-supplement. MALDI-TOF MS, Matrix-assisted laser desorption ionization-time of flight mass spectrometry. Figure created with BioRender.com.

All enriched samples (floor drainage samples and enriched wastewater) were spread on agar plates with a 10 μL loop. Raw, non-enriched wastewater samples were inoculated into CHROMagar ESBL, CHROMagar mSuperCARBA, and CHROMagar VRE (100 μL) or into CHROMagar MRSA and CHROMagar Candida Plus (333 μL). The culture of a larger volume (333 μL) on the latter two plates was based on previous experience. All plates were incubated at 37°C for 18–24 h, except the CHROMagar Candida Plus plates, which were incubated for up to 72 h (Figure 2).

Single bacterial colonies were selected from each plate based on colony morphology according to the manufacturer’s instructions. Up to five colonies representing typical colony morphologies for each targeted microbe were collected from each selective agar. Each colony was subcultured and purified on the corresponding chromogenic agar using a 1 μL sterile loop and incubated aerobically at 37°C for 18–24 h (CHROMagar Candida Plus up to 72 h). Purified isolates were subcultured on bovine blood agar plates (Columbia Blood Agar Base, Oxoid Ltd., Basingstoke, United Kingdom) and incubated aerobically for 18–24 h at 37°C for further characterization (Figure 2).

### 2.4 Identification

Bacterial species were identified by Matrix-assisted laser desorption ionization-time of flight mass spectrometry (MALDI-TOF MS) based on the Bruker Microflex LT/SH (Version BDAL 2021–05-26 T10:10:57.442) (Bruker Daltonics GmbH & Co. KG, Bremen, Germany). The criterion for MALDI-TOF MS bacterial identification was a score value of >2.0 which was considered high confidence, per the manufacturer’s instructions.

### 2.5 Antimicrobial susceptibility testing

The phenotype of all *E. coli* and *K. pneumoniae* isolates collected from CHROMagar ESBL was determined with a disk diffusion test according to the EUCAST (European Committee of Antimicrobial Susceptibility Testing) standard (17), with cefotaxime (5 μg) (Oxoid Ltd., Basingstoke, Hampshire, United Kingdom), ceftazidime (10 μg), cefoxitin (30 μg), cefepime (30 μg), and meropenem (10 μg) (Neo-Sensitabs, Rosco Diagnostica A/S, Taastrup, Denmark). The results were interpreted according to EUCAST epidemiological cut-off values (ECOFFs) (18). Synergism between third generation cephalosporins and clavulanic acid was also tested with a combination disk diffusion test, using cefotaxime 30 μg + clavulanic acid 10 μg and ceftazidime 30 μg + clavulanic acid 10 μg (Neo-Sensitabs, Rosco Diagnostica A/S, Taastrup, Denmark). *E. coli* ATCC 25922 was used as the quality control strain.

### 2.6 DNA extraction, whole genome sequencing (WGS), and bioinformatic analyses

A subset of *E. coli* (*n* = 15) and *K. pneumoniae* (*n* = 6) isolates from CHROMagar ESBL were subjected to whole genome sequencing (WGS) with the following criteria: 1 isolate of each species from each floor drainage and wastewater sample from each abattoir, where applicable. Strains were grown in Tryptone Soya Broth (Oxoid, Basingstoke, United Kingdom) at 37°C for 16–18 h. DNA was extracted from cells harvested from 1 mL of culture by using QIAcube Connect instrument (QIAGEN, Hilden, Germany) with DNeasy Blood & Tissue kit (QIAGEN, Valencia, CA, United States). DNA quantity was measured using a Qubit 2.0 fluorometer (Invitrogen, Life Technologies, Carlsbad, CA, United States). The quality of DNA was assessed by using a NanoDrop ND-1000 spectrophotometer (Thermo Fischer Scientific, Wilmington, DE, United States) based on the 260/280 ratio. Library preparation was performed with a NEBNext Ultra DNA Library Prep Kit for Illumina with 300 bp fragment length. Sequencing was performed with Illumina NovaSeq 6,000 (outsourced to Novogene, Cambridge, United Kingdom) with targeted genomic coverage of 100× and 2 × 150 bp read length.

All sequenced isolates were analyzed with Ridom SeqSphere+ software v7.7.5 (Ridom GmbH, Germany) (19). Quality analysis of the sequences was performed with FastQC v0.1.1.7 (20), and adapters were removed with Trimmomatic v0.36 (21). Raw reads were assembled with SKESA v2.3.0 using default settings (22). Quality trimming was performed with an average quality of ≥30 and a window of 20 bases. Remapping and polishing were performed with the BWA-MEM mapping algorithm. Sequencing statistics are presented in Supplementary Table S1. Species identification was done with Mash Distance (23). Species of one isolate was confirmed with KmerFinder 3.2 (24–26) through the Center for Genomic Epidemiology web server (DTU, Denmark). Acquired AMR genes were identified from assembled genomes with NCBI AMRFinderPlus 3.2.3 (27), using 100% alignment and > 90% identity. ResFinder 4.5.0 was used through the Center for Genomic Epidemiology web server (DTU, Denmark) to determine the allelic variants of beta-lactamase genes (*bla*) (28, 29). STs were analyzed by using multi-locus sequence typing (MLST) (30). The Warwick MLST scheme was chosen for *E. coli* isolates. *E. coli* isolates with novel STs were submitted to Enterobase (31). *E. coli* serotypes were identified with Center for Genomic Epidemiology SerotypeFinder (32) while virulence factors were searched for with VFDB (33). Plasmid replicons carried by the studied isolates were determined from assembled genomes with PladmidFinder 2.1 (29, 34), with an identity threshold of 95% and a minimum length of 60%. Phylogenetic analysis was conducted for all *E. coli* and *K. pneumoniae* isolates with core genome multilocus sequence typing (cgMLST) by comparing 2,513 and 2,358 alleles, pairwise ignoring missing values, respectively.

## 3 Results

### 3.1 Bacterial species identified in the abattoir floor drainage swabs, and wastewater and antimicrobial susceptibility

In total, 366 isolates were selected from the initial agar plates, with up to 12 isolates per sample. Among the isolates that withstood MALDI-TOF MS (*n* = 314/366), 286 were identified, revealing 37 different bacterial species (Supplementary Table S2). Out of all identified species, *E. coli* (*n* = 60) and *K. pneumoniae* (*n* = 24) were the most abundant, both identified solely on CHROMagar ESBL. *A. baumannii* (*n* = 16), *Lysinibacillus xylanilyticus* (*n* = 22), *Enterococcus gallinarum* (*n* = 22), and *Myerozyma guilliermondii* (*n* = 10) were the most abundant species identified on CHROMagar mSuperCARBA, CHROMagar MRSA, CHROMagar VRE, and CHROMagar CandidaPlus, respectively. MRSA*, P. aeruginosa*, Vancomycin-resistant *Enterococcus* (VRE), or *C. auris* were not identified. All identified species in different samples, abattoirs, and growth media are presented in Supplementary Table S2.

ESBL *E. coli* was detected in the floor drainage samples of five (83.3%) abattoirs, and in wastewater samples of two (33%) abattoirs, whereas *K. pneumoniae* was detected solely in the floor drainage samples of three (50%) abattoirs. Neither *E. coli* nor *K. pneumoniae* were detected in abattoir 4, which slaughters cattle, sheep, and pigs (Supplementary Table S2).

All *E. coli* (*n =* 60) and *K. pneumoniae* (*n =* 24) isolates exhibited resistance against cefotaxime and cefepime and were susceptible to cefoxitin, representing ESBL-phenotype. One *E. coli* isolate exhibited resistance against meropenem. Antimicrobial resistance profiles of the isolates are presented in Supplementary Table S3.

### 3.2 Multi-locus sequence types (MLST), antimicrobial resistance genes, phylogenetics, serotypes, and virulence factors

**Figure 3:**
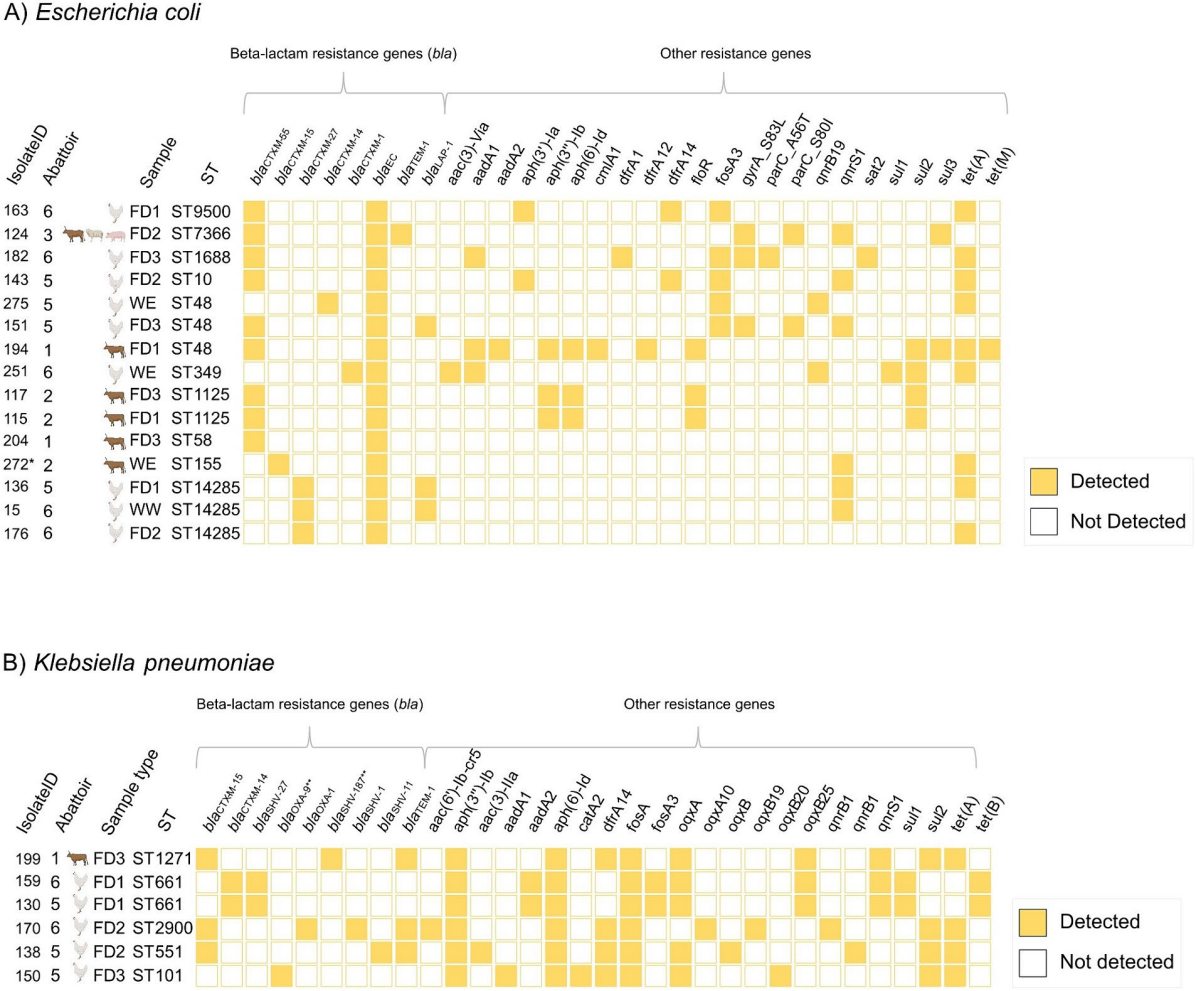
Sequence types (ST), beta-lactamase genes, and resistance genes other than beta-lactamases for the whole-genome sequenced isolates of **(A)** *Escherichia coli* (*n* = 15) and **(B)** *Klebsiella pneumoniae* (*n* = 6). Filled squares indicate the presence of the resistance gene. Abattoirs 1–6 indicated with number. Isolate ID, Isolate identification code. FD1–3, Floor drainage sample 1–3. WE, enriched wastewater sample. WW, wastewater sample. ST, sequence type. *Species confirmed with KmerFinder 3.2. **The allelic variants of beta-lactamase genes (*bla*) identified with ResFinder 4.5.0. iTOL (https://itol.embl.de/) and Inkscape 1.3.2 were used for data visualization.

Ten different *E. coli* STs were identified. The most common *E. coli* STs were ST48 (*n* = 3/15, 20.0%) and ST14285 (*n* = 3/15, 20.0%). ST48 was detected in three abattoirs, while ST14285 was detected in two abattoirs. Two of the isolates (*n* = 2/15, 13.3%) were ST1125, both detected in the same abattoir. The remaining STs, which included ST10, ST155, ST1688, ST349, ST58, ST7366, and ST9500 were represented by single isolates only. Furthermore, five *K. pneumoniae* STs were identified. The most common was ST661 (*n* = 2/6, 33.3%). Other STs (ST101, ST1271, ST2900, and ST551) were present in single isolates only (Figure 3).

The most common ESBL-genes among *E. coli* were *bla*_CTX-M-55_ (*n* = 9/15, 60.0%) and *bla*_CTX-M-27_ (*n* = 3/15, 20.0%) while for *K. pneumoniae,* the most common ESBL-gene was *bla*_CTX-M-15_ (*n* = 3/6, 50.0%). Two (*n* = 2/6, 33.3%) *K. pneumoniae* isolates possessed both *bla*_CTX-M-14_ and *bla*_SHV-27_ concurrently. ESBL-genes were not identified in one *K. pneumoniae* isolate. All isolates carried at least one other beta-lactamase gene, such as *bla*_OXA-1_ and *bla*_EC_. Additionally, isolates carried up to 11 AMR genes other than beta-lactamase. All *K. pneumoniae* isolates and nine (*n* = 9/15, 60.0%) *E. coli* isolates harbored genes associated with tetracycline resistance (Figure 3). The majority of the sequenced *E. coli* isolates (*n* = 12/15, 80.0%) and all *K. pneumoniae* isolates were multidrug resistant (i.e., possessed genes encoding resistance to three or more antimicrobial classes (35)). No colistin or carbapenem resistance encoding genes were identified (Figure 3).

**Figure 4:**
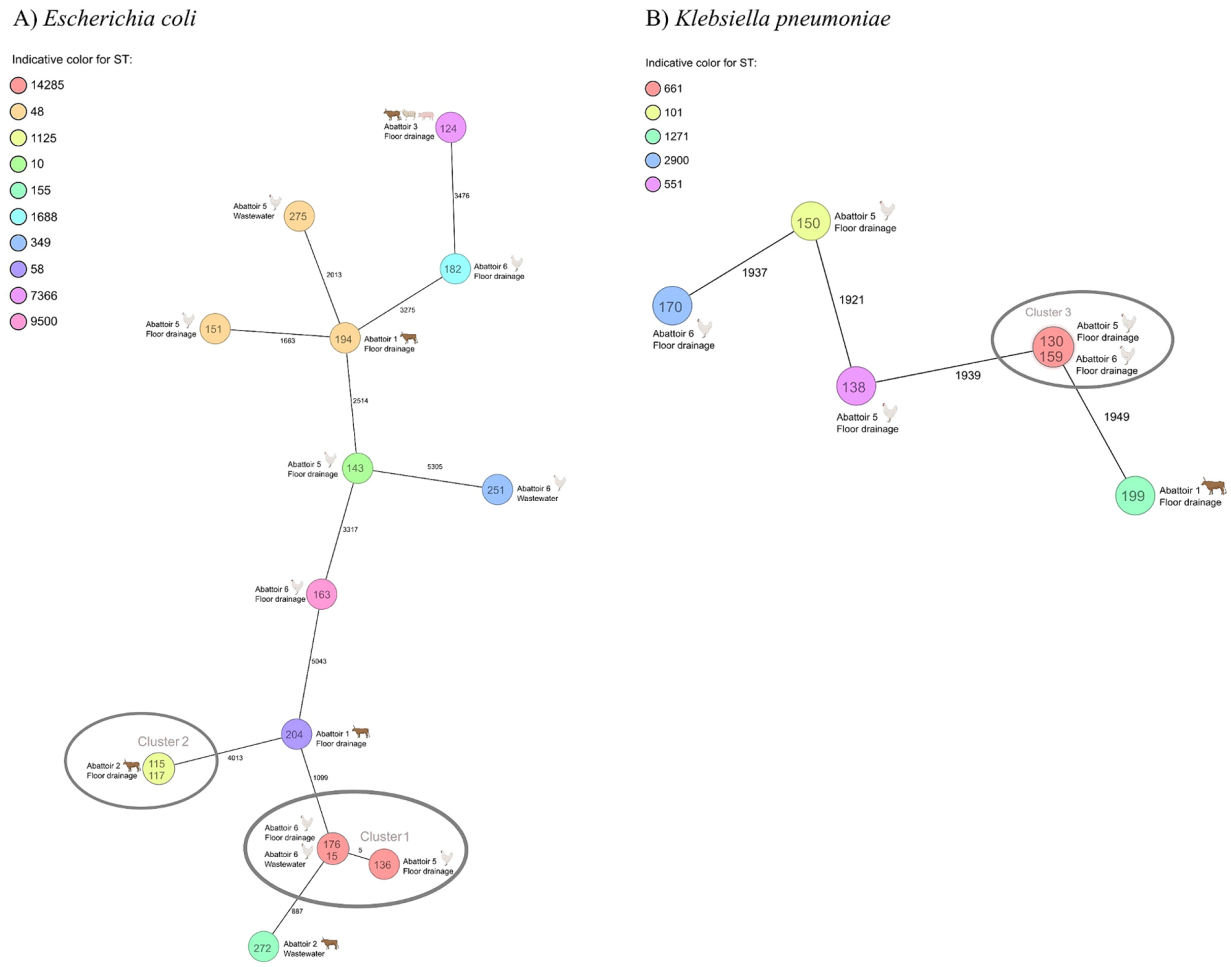
A minimum spanning tree of core genome multilocus sequence typing (cgMLST) of **(A)** ESBL-producing *Escherichia coli* isolates (*n* = 15) and **(B)** *Klebsiella pneumoniae* isolates (*n* = 6) from abattoir floor drainages and wastewater. Each circle represents one or multiple identical sequences. The numbers between the circles indicates the allele differences. Colors indicate sequence type. Text inside the circles indicate the isolate identification number, and text and figure next to the circles indicate the abattoir, sample type, and animal species handled in the abattoir. Clusters are indicated with gray circles. cgMLST for *Escherichia coli* isolates was based on 2,513 columns, pairwise ignoring missing values. CgMLST for *Klebsiella pneumoniae* isolates was based on 2,365 columns, pairwise ignoring missing values. ST, sequence type.

A total of 12 *E. coli* serotypes were identified. The most common *E. coli* serotype was O21:H51 (*n* = 3/15, 20.0%), followed by O139:H19 (*n* = 2/15, 13.3%). All *E. coli* serotypes are presented in Supplementary Table S4. None of the *E. coli* isolates harbored genes associated with Shiga toxin-production (*stx*). All identified virulence genes are presented in Supplementary Table S4. Phylogenetic analysis revealed closely related strains (<10 allele difference (36)) for *E. coli* and *K. pneumoniae. E. coli* isolates with ST14285 originating from the floor drainage and wastewater samples of poultry abattoirs (abattoirs 5 and 6) were closely related. Furthermore, two *E. coli* isolates with ST1125 originating from floor drainage samples of a cattle abattoir were closely related. For *K. pneumoniae,* two isolates with ST611 originating from the poultry abattoirs (abattoirs 5 and 6) were closely related. Minimum spanning tree presents the allelic differences between the *E. coli* (Figure 4A) and *K. pneumoniae* isolates (Figure 4B).

All isolates, except one *E. coli* isolate, were harboring at least two plasmid replicons. The most common plasmid replicon was IncFII, carried by 14 *E. coli* (93.3%) isolates and five (83.3%) *K. pneumoniae* isolates. All identified plasmid replicons are presented in Supplementary Table S4.

## 4 Discussion

In this pilot study, we describe key AMR pathogens, including ESBL-producing *E. coli* and *K. pneumoniae,* in the floor drainage and wastewater samples of the studied abattoirs. ESBL *E. coli* was detected in cattle, sheep, pig, and poultry abattoirs. ESBL *E. coli* was more frequently detected in floor drainages than in wastewater samples and was observed in five abattoirs. On the other hand, ESBL *K. pneumoniae* was less frequent and detected only in floor drainage samples in three abattoirs that slaughtered cattle or poultry. A previous study reported that ESBL *E. coli* is more common than ESBL *K. pneumoniae* in cattle (37). However, current data on ESBL *K. pneumoniae* in food-producing animals remains scarce and sporadic compared to the plethora of studies investigating the presence of ESBL *E. coli* in food-producing animals in many countries (38–45). A larger sample size, accompanied by statistical analysis, would be needed to comprehensively rule out coincidence and show whether *E. coli* is truly more frequent than *K. pneumoniae* in abattoirs.

STs and enzyme types detected in abattoir floor drainages and wastewater were similar to those described in food-producing animals in the literature (39–45). We identified a great diversity of STs, in accordance with previous studies showing high variability among ESBL-producing *E. coli* and *K. pneumoniae* (39, 42). Here, *E. coli* showed multiple STs, including ST48, ST10, ST155, and ST58. These STs have been previously reported in food-producing animals in several African countries, including South Africa, where ESBL *E. coli* ST10 was observed in pigs (39–45). A proportion of the detected *E. coli* STs, including ST48, ST10, ST349, and ST58, has also been occasionally reported in humans (46–49). *K. pneumoniae* showed five STs, including ST101 and ST661. ST101 and ST661 are linked to human disease outbreaks (50) and have also been reported in food-producing animals in Europe (51).

Genes belonging to the CTX-M-group were the most common ESBLs in the abattoir samples, with *bla*_CTX-M-55_ being the most prevalent among *E. coli* isolates, observed across five abattoirs. Globally, *bla*_CTX-M-55_ is the most common and widely distributed CTX-M-enzyme in *E. coli* isolates from food-producing animals (52). Previous studies have shown that the majority of ESBL *E. coli* isolated from dairy cattle in South Africa harbored CTX-M genes (53), but data on *bla*_CTX-M-55_ from Africa, including South Africa, is scanty. However, *bla*_CTX-M-55_ has been reported in ESBL *E. coli* from cattle in Nigeria (40). Interestingly, we detected *bla*_CTX-M-55_ in *E. coli* ST10, ST48, and ST58, consistent with previous studies showing that *bla*_CTX-M-55_ is prevalent in clones associated with animals (52). Another prevalent ESBL gene in food-producing animals is *bla*_CTX-M-15_ (40–42). In this study, *bla*_CTX-M-15_ was detected in an *E. coli* isolate from a cattle abattoir and in three *K. pneumoniae* isolates originating from three abattoirs slaughtering cattle or poultry.

We detected genetically closely related bacterial strains (<10 allele difference (36)) between two poultry abattoirs. This observation suggests that abattoir samples mirror the microbiota of the food-producing animals. In the pyramidal poultry production system, birds in multiple production-level farms originate from the same hatchery and parental flock (54). It has been shown that ESBL *E. coli* may transfer to the production-level birds from the parental flock or the hatchery environment, and as a result, birds in multiple production-level farms can share similar microbiota (55, 56). As we did not collect samples simultaneously from the animals and abattoir environment and wastewater, we are not able to comprehensively prove that the isolates originated from the animals. However, it is difficult to constitute an alternative explanation for the clonality of the strains in two different abattoirs.

We recognize the various limitations of this pilot study. Here, simultaneous samples from the abattoirs and animals were not collected. Hence, we can only assume, but not conclusively prove, that the described isolates originated from the animals. Moreover, we identified species, such as *Pseudomonas* spp. and *Vagococcus* spp., that are less likely associated with the animals and more plausible with environmental sources. The fundamental challenge that wastewater surveillance studies still aim to answer is defining to what extent the samples represent the microbes originating from the studied host and how much noise microbes from the environment or sewage network biofilms are causing (57).

Revision and improvement of methodological factors are also necessary. Here, wastewater was collected as grab samples, whereas composite samples collected with passive collectors could be less susceptible to the effects of changes in wastewater flow rates (58). Freezing the samples before culturing may have also influenced the bacterial composition. Hence, handling and analyzing the samples locally would reduce the potential effects of pre-handling, freezing, and shipping. The enrichment protocols could be further optimized for each targeted microbe. For example, this study used BPW, whereas, for MRSA, Müeller-Hinton broth with 6.5% sodium chloride could be more suitable (59). Noteworthy, the quantitation of the bacteria is not possible from the enriched samples, and using only selective culturing does not enable defining the overall resistance rate as the ratio between resistant and susceptible isolates cannot be evaluated. In the EU, the phenotypical resistance rates in indicator *E. coli* in food-producing animals are monitored (60). A similar methodology could be potentially utilized in WES, but the data should be interlinked with similar data from food-producing animals to interpret the results. In EU surveillance, the broth microdilution method is used to determine the antimicrobial susceptibility of food-producing animal isolates and congruent methods should be used if the phenotypical susceptibility results are compared between animals and WES. There is no recurrent surveillance data from South Africa to interlink the data from this study, and we used a disk diffusion test only to confirm the phenotype of the isolates. To address the gaps in this study and diminish some of the challenges associated with WES, such as associating the detected microbes with the host, we suggest that WES could be further studied in countries where routine AMR surveillance in food-producing animals exists. Alternatively, long-term studies could be structured to obtain simultaneous samples from the animals and abattoir environment and wastewater.

In the future, it should be acknowledged that commercial selective culture media come with relatively high expenses, limiting their introduction to recurrent surveillance. Therefore, other options could be considered. For example, MacConkey with cefotaxime is less expensive and is already a validated method in the EU for monitoring ESBL-and AmpC-producing *E. coli* in meat and cecal samples of animals (60). Genotypic data, such as STs and resistance gene profiles are valuable from an epidemiological standpoint. WGS and new, continuously evolving DNA sequencing technologies can extract valuable data from complex WES samples, but PCR-based methods could also provide relevant information if the resources do not enable the use of these technologies.

The abattoir structures should be evaluated thoroughly, and a comprehensive study comparing samples from different locations should be conducted to identify optimal sampling points in abattoirs and to minimize the risk of disruption of human-origin microbiota. Abattoir wastewater tanks consist of wastewater originating from various sources, including water used during the slaughter process and cleaning, but, depending on the abattoirs, may also include wastewater from staff premises. Here, the results suggest focusing the sampling at the beginning of the pipeline, i.e., in floor drainages, instead of wastewater tanks. A previous study showing a higher proportion of host-associated microbes at the beginning of the pipeline (61) also supports this observation. Moreover, the abundance of microbes originating from animals is likely higher on the dirty processing side, i.e., barn, stunning, and skinning, which would advocate for focusing the sampling in this area.

Animals, particularly food-producing animals, have a significant role in AMR. AMR surveillance of food-producing animals has substantial relevance for public health as it helps to understand the dynamics of AMR pathogens and their transmission between animals, humans, and the environment. Collecting a representative number of samples is laborious and expensive, and limited resources are one of the main challenges in AMR surveillance of food-producing animals. New perspectives could help to improve global AMR surveillance in this sector. Wastewater surveillance of AMR is not yet well-established, but wastewater is still an underutilized resource in many sectors. This pilot study shows that clones of AMR bacteria known to be circulating in the animals can be detected by WES and advocates for future discussion and research related to WES in the veterinary sector. Interpreting the results could be more straightforward by optimizing the methodology, for example, by studying non-enriched samples, using various protocols for enrichment depending on the targeted microbes, and culturing the samples directly after collection. Replicating this study with an optimized methodology, including samples collected simultaneously from the animals or comparing into previously collected samples from the same country, could provide substantial information about the feasibility of abattoir WES for AMR surveillance of food-producing animals. WES could offer a less resource-consuming methodology for AMR surveillance of food-producing animals, which would cause minimal disturbance for the abattoir production line with no intervention with animals or carcasses. Hence, the sampling is more flexible, and there is no need to synchronize the sampling with the speed of the production line. Furthermore, this approach could help implement AMR surveillance of food-producing animals, also in LMICs.

## Data Availability

The datasets presented in this study can be found in online repositories. The names of the repository/repositories and accession number(s) can be found in the article/Supplementary material.
