# Supplementary material for: Wastewater and environmental sampling holds potential for antimicrobial resistance surveillance in food-producing animals - a pilot study in South African abattoirs
Source: Front Vet Sci. 2024 Oct 3;11:1444957. doi: 10.3389/fvets.2024.1444957 (PMC11483616; doi:10.3389/fvets.2024.1444957)
Supplement: Supplementary file 3 [file Table_3.docx]

*Supplementary Table 3. Zone of inhibition of antimicrobials for 60 Escherichia coli and 24 Klebsiella pneumoniae isolated from CHROMagar Orientation with ESBL-supplement. Samples were obtained from floor drainages (FD) and wastewater (WW) of six South African abattoirs in 2022.*

| Abattoir | Sample type | **Isolate ID** | **Species** | **CTX 5** | **CAZ 10** | **MRP 10** | **CFO 30** | **FEP 30** | **CTX 30** | **CTX +C** | **CAZ 30** | **CAZ +C** | **Difference CTX30/ CTX+C** | **Difference CAZ30/ CAZ+C** | **Pheno-type** |
| --- | --- | --- | --- | --- | --- | --- | --- | --- | --- | --- | --- | --- | --- | --- | --- |
| ECOFFs (mm)  *E. coli/K. pneumoniae* |  |  |  | 22  /21 | 21/  20 | 26  /24 | 18  /18 | 28  /26 |  |  |  |  |  |  |  |
| 1 | FD1 | **194** | *E. coli* | 6 | 14 | 32 | 23 | 19 | 11 | 29 | 17 | 29 | 18 | 12 | ESBL |
|  |  | 195 | *E. coli* | 6 | 14 | 32 | 23 | 17 | 10 | 29 | 15 | 29 | 19 | 14 | ESBL |
|  |  | 196 | *E. coli* | 6 | 14 | 31 | 23 | 18 | 10 | 29 | 16 | 28 | 19 | 12 | ESBL |
|  |  | 197 | *E. coli* | 6 | 14 | 31 | 22 | 17 | 10 | 30 | 15 | 29 | 20 | 14 | ESBL |
|  |  | 198 | *E. coli* | 6 | 15 | 33 | 23 | 18 | 10 | 29 | 15 | 29 | 19 | 14 | ESBL |
|  | FD3 | **199** | *K. pneumoniae* | 6 | 12 | 30 | 24 | 16 | 10 | 30 | 13 | 28 | 20 | 15 | ESBL |
|  |  | 200 | *K. pneumoniae* | 6 | 12 | 31 | 23 | 17 | 11 | 31 | 13 | 28 | 20 | 15 | ESBL |
|  |  | 201 | *K. pneumoniae* | 6 | 11 | 31 | 24 | 16 | 10 | 30 | 13 | 30 | 20 | 17 | ESBL |
|  |  | 202 | *K. pneumoniae* | 6 | 12 | 32 | 24 | 18 | 11 | 30 | 14 | 29 | 19 | 15 | ESBL |
|  |  | 203 | *K. pneumoniae* | 6 | 12 | 32 | 23 | 16 | 10 | 30 | 14 | 29 | 20 | 15 | ESBL |
|  |  | **204** | *E. coli* | 6 | 15 | 33 | 25 | 18 | 11 | 31 | 17 | 31 | 20 | 14 | ESBL |
|  |  | 205 | *E. coli* | 6 | 16 | 30 | 24 | 19 | 12 | 30 | 17 | 28 | 18 | 11 | ESBL |
|  |  | 206 | *E. coli* | 6 | 15 | 30 | 23 | 17 | 11 | 30 | 17 | 29 | 19 | 12 | ESBL |
|  |  | 207 | *E. coli* | 6 | 16 | 29 | 24 | 15 | 11 | 33 | 17 | 32 | 22 | 15 | ESBL |
|  |  | 208 | *E. coli* | 6 | 17 | 30 | 24 | 18 | 12 | 31 | 17 | 31 | 19 | 14 | ESBL |
| 2 | FD1 | **115** | *E. coli* | 6 | 14 | 31 | 23 | 15 | 9 | 30 | 16 | 29 | 21 | 13 | ESBL |
|  |  | 116 | *E. coli* | 6 | 8 | 31 | 23 | 15 | 10 | 30 | 16 | 28 | 20 | 12 | ESBL |
|  | FD3 | **117** | *E. coli* | 6 | 12 | 33 | 23 | 17 | 11 | 30 | 15 | 29 | 19 | 14 | ESBL |
|  |  | 118 | *E. coli* | 6 | 14 | 33 | 22 | 17 | 10 | 32 | 15 | 29 | 22 | 14 | ESBL |
|  |  | 119 | *E. coli* | 6 | 14 | 32 | 22 | 17 | 10 | 32 | 15 | 30 | 22 | 15 | ESBL |
|  |  | 120 | *E. coli* | 6 | 14 | 33 | 23 | 17 | 10 | 32 | 15 | 30 | 22 | 15 | ESBL |
|  |  | 121 | *E. coli* | 6 | 16 | 33 | 24 | 20 | 12 | 33 | 16 | 31 | 21 | 15 | ESBL |
|  | WWe | **272** | *E. coli* | 10 | 19 | 31 | 24 | 22 | 17 | 31 | 21 | 29 | 14 | 8 | ESBL |
| 3 | FD2 | **124** | *E. coli* | 6 | 13 | 31 | 22 | 18 | 10 | 30 | 15 | 28 | 20 | 13 | ESBL |
|  |  | 125 | *E. coli* | 6 | 13 | 31 | 23 | 17 | 9 | 30 | 15 | 28 | 21 | 13 | ESBL |
| 5 | FD1 | **130** | *K. pneumoniae* | 6 | 21 | 29 | 23 | 18 | 14 | 29 | 23 | 30 | 15 | 7 | ESBL |
|  |  | 131 | *K. pneumoniae* | 6 | 22 | 30 | 23 | 19 | 13 | 29 | 23 | 30 | 16 | 7 | ESBL |
|  |  | 132 | *K. pneumoniae* | 6 | 22 | 26 | 22 | 18 | 15 | 29 | 23 | 28 | 14 | 5 | ESBL |
|  |  | 133 | *E. coli* | 6 | 13 | 30 | 22 | 17 | 10 | 32 | 17 | 32 | 22 | 15 | ESBL |
|  |  | 134 | *E. coli* | 6 | 19 | 31 | 21 | 16 | 12 | 29 | 19 | 28 | 17 | 9 | ESBL |
|  |  | 135 | *E. coli* | 6 | 18 | 33 | 25 | 19 | 12 | 34 | 20 | 32 | 22 | 12 | ESBL |
|  |  | **136** | *E. coli* | 6 | 16 | 28 | 20 | 18 | 12 | 29 | 17 | 28 | 17 | 11 | ESBL |
|  |  | 137 | *E. coli* | 6 | 12 | 28 | 22 | 7 | 11 | 23 | 14 | 27 | 12 | 13 | ESBL |
|  | FD2 | **138** | *K. pneumoniae* | 6 | 12 | 31 | 24 | 15 | 9 | 30 | 14 | 29 | 21 | 15 | ESBL |
|  |  | 139 | *K. pneumoniae* | 6 | 12 | 30 | 23 | 17 | 9 | 31 | 15 | 29 | 22 | 14 | ESBL |
|  |  | 140 | *K. pneumoniae* | 6 | 13 | 26 | 22 | 17 | 9 | 31 | 14 | 29 | 22 | 15 | ESBL |
|  |  | 141 | *K. pneumoniae* | 6 | 13 | 30 | 22 | 17 | 11 | 30 | 15 | 28 | 19 | 13 | ESBL |
|  |  | 142 | *K. pneumoniae* | 6 | 12 | 30 | 21 | 17 | 9 | 31 | 13 | 29 | 22 | 16 | ESBL |
|  |  | **143** | *E. coli* | 6 | 18 | 33 | 24 | 20 | 11 | 33 | 19 | 32 | 22 | 13 | ESBL |
|  |  | 144 | *E. coli* | 7 | 20 | 28 | 23 | 19 | 12 | 28 | 22 | 28 | 16 | 6 | ESBL |
|  |  | 145 | *E. coli* | 6 | 12 | 27 | 18 | 15 | 9 | 28 | 13 | 28 | 19 | 15 | ESBL |
|  |  | 146 | *E. coli* | 6 | 15 | 30 | 24 | 18 | 10 | 30 | 15 | 30 | 20 | 15 | ESBL |
|  |  | 147 | *E. coli* | 6 | 13 | 28 | 17 | 16 | 10 | 27 | 13 | 27 | 17 | 14 | ESBL |
|  | FD3 | **150** | *K. pneumoniae* | 12 | 9 | 33 | 22 | 25 | 17 | 32 | 10 | 29 | 15 | 19 | ESBL |
|  |  | **151*** | *E. coli* | 6 | 16 | 20 | 20 | 15 | 9 | 31 | 10 | 31 | 22 | 21 | ESBL |
|  |  | 152 | *E. coli* | 6 | 14 | 33 | 21 | 17 | 10 | 32 | 14 | 29 | 22 | 15 | ESBL |
|  |  | 153 | *E. coli* | 6 | 13 | 28 | 19 | 16 | 10 | 30 | 13 | 28 | 20 | 15 | ESBL |
|  |  | 154 | *E. coli* | 6 | 14 | 32 | 22 | 16 | 10 | 31 | 14 | 28 | 21 | 14 | ESBL |
|  |  | 155 | *E. coli* | 6 | 20 | 33 | 21 | 17 | 12 | 30 | 20 | 29 | 18 | 9 | ESBL |
|  | WWe | **275** | *E. coli* | 7 | 23 | 33 | 25 | 21 | 16 | 30 | 25 | 31 | 14 | 6 | ESBL |
|  |  | 276 | *E. coli* | 7 | 23 | 33 | 25 | 20 | 15 | 30 | 26 | 31 | 15 | 5 | ESBL |
|  |  | 277 | *E. coli* | 9 | 23 | 32 | 25 | 20 | 15 | 31 | 24 | 30 | 16 | 6 | ESBL |
|  |  | 278 | *E. coli* | 9 | 23 | 28 | 25 | 22 | 14 | 31 | 23 | 30 | 17 | 7 | ESBL |
|  |  | 279 | *E. coli* | 6 | 22 | 31 | 23 | 21 | 15 | 32 | 24 | 31 | 17 | 7 | ESBL |
| 6 | FD1 | 158 | *K. pneumoniae* | 6 | 22 | 30 | 23 | 20 | 14 | 31 | 22 | 29 | 17 | 7 | ESBL |
|  |  | **159** | *K. pneumoniae* | 6 | 22 | 31 | 23 | 21 | 14 | 28 | 24 | 29 | 14 | 5 | ESBL |
|  |  | 160 | *K. pneumoniae* | 6 | 22 | 30 | 23 | 20 | 15 | 29 | 25 | 31 | 14 | 6 | ESBL |
|  |  | 161 | *K. pneumoniae* | 6 | 22 | 30 | 23 | 19 | 14 | 30 | 23 | 29 | 16 | 6 | ESBL |
|  |  | 162 | *K. pneumoniae* | 6 | 23 | 26 | 23 | 20 | 15 | 30 | 23 | 29 | 15 | 16 | ESBL |
|  |  | **163** | *E. coli* | 6 | 13 | 27 | 19 | 17 | 10 | 30 | 14 | 28 | 20 | 14 | ESBL |
|  |  | 164 | *E. coli* | 6 | 12 | 32 | 21 | 18 | 10 | 30 | 14 | 28 | 20 | 14 | ESBL |
|  |  | 165 | *E. coli* | 6 | 12 | 32 | 20 | 16 | 9 | 29 | 13 | 28 | 20 | 15 | ESBL |
|  |  | 166 | *E. coli* | 6 | 18 | 33 | 25 | 20 | 15 | 34 | 22 | 33 | 19 | 11 | ESBL |
|  |  | 167 | *E. coli* | 6 | 17 | 32 | 23 | 19 | 11 | 33 | 17 | 31 | 22 | 14 | ESBL |
|  | FD2 | **170** | *K. pneumoniae* | 6 | 12 | 26 | 23 | 16 | 9 | 30 | 13 | 29 | 21 | 16 | ESBL |
|  |  | 171 | *K. pneumoniae* | 14 | 9 | 30 | 22 | 24 | 21 | 33 | 10 | 28 | 12 | 18 | ESBL |
|  |  | 172 | *K. pneumoniae* | 6 | 12 | 29 | 21 | 17 | 9 | 30 | 13 | 29 | 21 | 16 | ESBL |
|  |  | 173 | *K. pneumoniae* | 6 | 13 | 27 | 22 | 19 | 10 | 29 | 15 | 31 | 19 | 16 | ESBL |
|  |  | 174 | *K. pneumoniae* | 14 | 9 | 27 | 22 | 25 | 21 | 32 | 10 | 28 | 11 | 18 | ESBL |
|  |  | 175 | *E. coli* | 6 | 13 | 28 | 20 | 16 | 11 | 30 | 14 | 28 | 19 | 14 | ESBL |
|  |  | **176** | *E. coli* | 6 | 16 | 28 | 19 | 18 | 12 | 28 | 18 | 28 | 16 | 10 | ESBL |
|  |  | 177 | *E. coli* | 6 | 12 | 28 | 18 | 14 | 11 | 27 | 14 | 27 | 16 | 13 | ESBL |
|  |  | 178 | *E. coli* | 6 | 13 | 27 | 18 | 16 | 9 | 30 | 14 | 28 | 21 | 14 | ESBL |
|  |  | 179 | *E. coli* | 6 | 12 | 28 | 19 | 15 | 10 | 29 | 13 | 28 | 19 | 15 | ESBL |
|  | FD3 | **182** | *E. coli* | 6 | 16 | 28 | 24 | 17 | 11 | 32 | 17 | 31 | 21 | 14 | ESBL |
|  |  | 183 | *E. coli* | 6 | 14 | 28 | 22 | 18 | 10 | 30 | 15 | 28 | 20 | 13 | ESBL |
|  |  | 184 | *E. coli* | 6 | 17 | 29 | 23 | 16 | 9 | 32 | 19 | 32 | 23 | 13 | ESBL |
|  |  | 185 | *E. coli* | 6 | 12 | 28 | 18 | 15 | 10 | 32 | 14 | 28 | 22 | 14 | ESBL |
|  | WW | **15** | *E. coli* | 6 | 18 | 33 | 20 | 20 | 11 | 32 | 19 | 30 | 21 | 11 | ESBL |
|  | WWe | **251** | *E. coli* | 6 | 22 | 33 | 23 | 19 | 14 | 31 | 23 | 31 | 17 | 8 | ESBL |
|  |  | 252 | *E. coli* | 6 | 22 | 32 | 24 | 17 | 12 | 30 | 23 | 31 | 18 | 8 | ESBL |
|  |  | 253 | *E. coli* | 6 | 22 | 32 | 23 | 17 | 14 | 32 | 23 | 30 | 18 | 7 | ESBL |
|  |  | 254 | *E. coli* | 6 | 21 | 32 | 23 | 17 | 13 | 32 | 22 | 30 | 19 | 8 | ESBL |
|  |  | 255 | *E. coli* | 6 | 21 | 32 | 24 | 17 | 12 | 32 | 23 | 30 | 20 | 7 | ESBL |

*Epidemiological cut-off values (ECOFFs) (mm) are indicated. ECOFFs before slash for E. coli and after slash for K. pneumoniae. Isolate ID (identification number) with bold lettering indicates that the isolate was subjected to whole-genome sequencing (n=21). FD1-3, Floor drainage sample 1-3. WW, Raw wastewater. WWe, Enriched wastewater. CTX 5, Cefotaxime 5 µg. CAZ 10, Ceftazidime 10 µg. MRP 10, Meropenem 10 µg. CFO 30, Cefoxitin 30 µg. FEP 30, Cefepime 30 µg. CTX 30, Cefotaxime 30 µg. CTX+C, Cefotaxime 30 µg + Clavulanic acid 10 µg. CAZ 30, Ceftazidime 30 µg. CAZ+C, Ceftazidime 30 µg + Clavulanic acid 10 µg. * Isolate 151 expressing resistance against meropenem.*
