# Supplementary material for: Wastewater and environmental sampling holds potential for antimicrobial resistance surveillance in food-producing animals - a pilot study in South African abattoirs
Source: Front Vet Sci. 2024 Oct 3;11:1444957. doi: 10.3389/fvets.2024.1444957 (PMC11483616; doi:10.3389/fvets.2024.1444957)
Supplement: Supplementary file 4 [file Table_4.docx]

Supplementary Table 4. Plasmid replicons and virulence genes carried by the Whole genome sequenced isolates of *Escherichia coli* (n=15) and *Klebsiella pneumoniae* (n=6) and serotypes of *Escherichia coli* (n=15) isolated from abattoir floor drainages and wastewater. Serotypes were determined from assembled genomes with Center for Genomic Epidemiology SerotypeFinder using ≥85% identity and ≥60% aligned overlap. Plasmid replicons were determined from assembled genomes with Center for Genomic Epidemiology PlasmidFinder 2.1 using ≥95% identity and ≥60% minimum coverage. Virulence genes were determined from assembled genomes with VFDB in Ridom SeqSphere+ using 100% alignment and ≥85% identity.

*Indicates an unknown O-serotype

| Species | Isolate | Abattoir | Origin | Serotype (for *E. coli)* | Plasmid replicons | Virulence genes |
| --- | --- | --- | --- | --- | --- | --- |
| *Escherichia coli* | 194 | 1 | Floor Drainage | O8:H11 | IncFII(pHN7A8), IncX1 | aslA, entB, entC, entD, entE, entS, espL1, espL4, espX1, espX4, espX5, fepA, fepB, fepC, fepD, fepG, fes, fimD, fimF, fimG, fimH, ompA, yagV/ecpE, yagW/ecpD, yagX/ecpC, yagY/ecpB, yagZ/ecpA, ykgK/ecpR |
| *Escherichia coli* | 204 | 1 | Floor Drainage | O*:H25 | IncFII(pHN7A8), IncN | entB, entC, entD, entE, entS, espL1, espX1, espX4, espX5, fdeC, fepA, fepB, fepC, fepD, fepG, fes, fimA, fimB, fimC, fimD, fimE, fimF, fimG, fimH, fimI, ompA, yagV/ecpE, yagW/ecpD, yagX/ecpC, yagY/ecpB, yagZ/ecpA, ykgK/ecpR |
| *Escherichia coli* | 115 | 2 | Floor Drainage | O129:H19 | IncFII, IncFII(pCoo),IncFII(pHN7A8), IncFIA, IncFIB(AP001918), IncN, IncR | astA, east1, entB, entC, entD, entE, entS, espL1, espX1, espX4, espX5, fdeC, fepA, fepB, fepC, fepD, fepG, fes, fimA, fimB, fimC, fimD, fimE, fimF, fimG, fimH, fimI, hlyA, hlyB, hlyD, ompA, yagV/ecpE, yagW/ecpD, yagX/ecpC, yagY/ecpB, yagZ/ecpA, ykgK/ecpR |
| *Escherichia coli* | 117 | 2 | Floor Drainage | O139:H19 | IncFII, IncFII(pCoo), IncFII(pHN7A8), IncFIA, IncFIB(AP001918), IncN, IncR | astA, east1, entB, entC, entD, entE, entS, espL1, espX1, espX4, espX5, fdeC, fepA, fepB, fepC, fepD, fepG, fes, fimA, fimB, fimC, fimD, fimE, fimF, fimG, fimH, fimI, hlyA, hlyB, hlyD, ompA, yagV/ecpE, yagW/ecpD, yagX/ecpC, yagY/ecpB, yagZ/ecpA, ykgK/ecpR |
| *Escherichia coli* | 272 | 2 | Wastewater | O*:H21 |  | entB, entC, entD, entE, entS, espL1, espX1, espX4, espX5, fepA, fepB, fepC, fepD, fepG, fes, ompA |
| *Escherichia coli* | 124 | 3 | Floor Drainage | O184:H30 | IncFII(29), IncFII(pHN7A8), IncFIB(AP001918), IncX1, IncI1-I(Alpha) | aslA, entB, entC, entD, entE, entS, espL1, espX1, espX4, espX5, fdeC, fepA, fepB, fepC, fepD, fepG, fes, fimA, fimB, fimC, fimD, fimE, fimF, fimG, fimH, fimI, ompA, yagV/ecpE, yagW/ecpD, yagX/ecpC, yagY/ecpB, yagZ/ecpA, ykgK/ecpR |
| *Escherichia coli* | 136 | 5 | Floor Drainage | O21:H51 | IncFII(pCoo), IncFIB(AP001918), IncN, IncI1-I(Alpha), IncY | entB, entC, entD, entE, entS, espL1, espX1, espX4, espX5, fdeC, fepA, fepB, fepC, fepD, fepG, fes, fimA, fimB, fimC, fimD, fimE, fimF, fimG, fimH, fimI, iroB, iroC, iroD, iroE, iroN, ompA, yagV/ecpE, yagW/ecpD, yagX/ecpC, yagY/ecpB, yagZ/ecpA, ykgK/ecpR |
| *Escherichia coli* | 143 | 5 | Floor Drainage | O12:H48 | IncFII, IncFII(pHN7A8), IncFIA, IncN, IncX1, Col440I | aslA, entB, entC, entD, entE, entS, espL1, espL4, espX4, espX5, fepA, fepB, fepC, fepD, fepG, fes, fimA, fimB, fimC, fimD, fimE, fimF, fimG, fimH, fimI, ompA, yagV/ecpE, yagW/ecpD, yagX/ecpC, yagY/ecpB, yagZ/ecpA, ykgK/ecpR |
| *Escherichia coli* | 151 | 5 | Floor Drainage | O88:H16 | IncFII(pHN7A8), IncFIB(K), p0111, Col440I | aslA, entB, entC, entD, entE, entS, espL1, espL4, espX1, espX4, fdeC, fepA, fepB, fepC, fepD, fepG, fes, fimA, fimB, fimC, fimD, fimE, fimF, fimG, fimH, fimI, kpsD, kpsM, ompA, yagV/ecpE, yagW/ecpD, yagX/ecpC, yagY/ecpB, yagZ/ecpA, ykgK/ecpR |
| *Escherichia coli* | 275 | 5 | Wastewater | O86:H10 | IncFII(29), IncI1-I(Alpha), p0111, Col440I, ColpVC, Col(pHAD28) | aslA, astA, east1, entB, entC, entD, entE, entS, espL1, espX1, espX4, espX5, fdeC, fepA, fepB, fepC, fepD, fepG, fes, fimA, fimB, fimC, fimD, fimE, fimF, fimG, fimH, fimI, kpsD, kpsM, ompA, yagV/ecpE, yagW/ecpD, yagX/ecpC, yagY/ecpB, yagZ/ecpA, ykgK/ecpR |
| *Escherichia coli* | 163 | 6 | Floor Drainage | O*:H32 | IncFII(pHN7A8), IncFIB(pLF82-PhagePlasmid), IncFIB(K), IncN, IncX1 | aslA, astA, east1, entB, entC, entD, entE, entS, espL1, espL4, espX4, espX5, fdeC, fepA, fepB, fepC, fepD, fepG, fes, fimF, fimG, fimH, ompA |
| *Escherichia coli* | 176 | 6 | Floor Drainage | O21:H51 | IncFII(pCoo), IncFIB(AP001918), IncN, IncI1-I(Alpha), IncY | entB, entC, entD, entE, entS, espL1, espX1, espX4, espX5, fdeC, fepA, fepB, fepC, fepD, fepG, fes, fimA, fimB, fimC, fimD, fimE, fimF, fimG, fimH, fimI, ompA, yagV/ecpE, yagW/ecpD, yagX/ecpC, yagY/ecpB, yagZ/ecpA, ykgK/ecpR |
| *Escherichia coli* | 182 | 6 | Floor Drainage | O*:H5 | IncFII, IncFII(pHN7A8), IncFIB(pLF82-PhagePlasmid), IncN, p0111, IncI1-I(Alpha), IncY | aslA, entB, entC, entD, entE, entS, espL1, espX1, espX4, espX5, fepA, fepB, fepC, fepD, fepG, fes, fimA, fimB, fimC, fimD, fimE, fimF, fimG, fimH, fimI, ompA, yagV/ecpE, yagW/ecpD, yagX/ecpC, yagY/ecpB, yagZ/ecpA, ykgK/ecpR |
| *Escherichia coli* | 15 | 6 | Wastewater | O21:H51 | IncFII(pCoo), IncFIB(AP001918), IncN, IncI1-I(Alpha) | entB, entC, entD, entE, entS, espL1, espX1, espX4, espX5, fdeC, fepA, fepB, fepC, fepD, fepG, fes, fimA, fimB, fimC, fimD, fimE, fimF, fimG, fimH, fimI, iroB, iroC, iroD, iroE, iroN, ompA, yagV/ecpE, yagW/ecpD, yagX/ecpC, yagY/ecpB, yagZ/ecpA, ykgK/ecpR |
| *Escherichia coli* | 251 | 6 | Wastewater | O3:H25 | IncFII, IncI1-I(Alpha), ColpVC, Col(pHAD28) | aslA, chuA, chuS, chuT, chuU, chuV, chuW, chuX, chuY, entB, entC, entD, entE, entS, espL1, espL4, espR1, espX1, espX2, espX4, espX5, espY2, espY3, espY4, fdeC, fepA, fepB, fepC, fepD, fepG, fes, fimA, fimB, fimC, fimD, fimE, fimF, fimG, fimH, fimI, ompA, yagV/ecpE, yagW/ecpD, yagX/ecpC, yagY/ecpB, yagZ/ecpA, ykgK/ecpR |
| *Klebsiella pneumoniae* | 199 | 1 | Floor drainage |  | IncFII(pKP91), IncFIA(pBK30683), IncFIB(K), Col440II | acrA, acrB, clpV/tssH, cpsACP, dotU/tssL, entA, entB, entC, entE, entF, fepA, fepB, fepC, fepD, fepG, fes, fimA, fimB, fimC, fimD, fimE, fimF, fimG, fimH, fimI, fimK, galF, glf, gnd, hcp/tssD, impA/tssA, iroE, kfoC, manB, manC, mrkA, mrkB, mrkC, mrkD, mrkF, mrkH, mrkI, mrkJ, rcsA, rcsB, sciN/tssJ, tssF, tssG, ugd, vasE/tssK, vgrG/tssI, vipA/tssB, vipB/tssC, wbbM, wbbN, wbbO, wzi, wzm, wzt, ybdA |
| *Klebsiella pneumoniae* | 130 | 5 | Floor drainage |  | IncFII(K), IncR | acrA, acrB, clpV/tssH, cpsACP, dotU/tssL, entA, entB, entC, entE, entF, fepA, fepB, fepC, fepD, fepG, fes, fimA, fimB, fimC, fimD, fimE, fimF, fimG, fimH, fimI, fimK, galF, glf, gnd, hcp/tssD, iroE, manB, manC, mrkA, mrkB, mrkC, mrkD, mrkF, mrkH, mrkI, mrkJ, rcsA, rcsB, sciN/tssJ, tssF, tssG, ugd, vasE/tssK, vipA/tssB, vipB/tssC, wbbM, wbbN, wbbO, wzi, wzm, wzt, ybdA |
| *Klebsiella pneumoniae* | 138 | 5 | Floor drainage |  | IncFIB(K), IncFIB(pKPHS1) | acrA, acrB, clpV/tssH, cpsACP, dotU/tssL, entA, entB, entC, entE, entF, fepA, fepB, fepC, fepD, fepG, fes, fimA, fimB, fimC, fimD, fimE, fimF, fimG, fimH, fimI, fimK, galF, glf, gnd, hcp/tssD, iroE, manB, manC, mrkA, mrkB, mrkC, mrkD, mrkF, mrkH, mrkI, mrkJ, rcsA, rcsB, sciN/tssJ, tssF, tssG, ugd, vasE/tssK, wbbM, wbbN, wbbO, wzi, wzm, wzt, ybdA |
| *Klebsiella pneumoniae* | 150 | 5 | Floor drainage |  | IncFII(pKP91), IncFIA(HI1), IncFIB(K), IncR, repB(R1701) | acrA, acrB, clpV/tssH, cpsACP, dotU/tssL, entA, entB, entC, entE, entF, fepA, fepB, fepC, fepD, fepG, fes, fimA, fimB, fimC, fimD, fimE, fimF, fimG, fimH, fimI, fimK, galF, glf, gnd, hcp/tssD, hcp/tssD, iroE, kfoC, mrkA, mrkB, mrkC, mrkD, mrkF, mrkH, mrkI, mrkJ, rcsA, rcsB, sciN/tssJ, tssF, tssG, ugd, vasE/tssK, wbbM, wbbN, wbbO, wzi, wzm, wzt, ybdA |
| *Klebsiella pneumoniae* | 159 | 6 | Floor drainage |  | IncFII(K), IncR | acrA, acrB, clpV/tssH, cpsACP, dotU/tssL, entA, entB, entC, entE, entF, fepA, fepB, fepC, fepD, fepG, fes, fimA, fimB, fimC, fimD, fimE, fimF, fimG, fimH, fimI, fimK, galF, glf, gnd, hcp/tssD, iroE, manB, manC, mrkA, mrkB, mrkC, mrkD, mrkF, mrkH, mrkI, mrkJ, rcsA, rcsB, sciN/tssJ, tssF, tssG, ugd, vasE/tssK, vipA/tssB, vipB/tssC, wbbM, wbbN, wbbO, wzi, wzm, wzt, ybdA |
| *Klebsiella pneumoniae* | 170 | 6 | Floor drainage |  | IncFII(K), IncFIB(K) | acrA, acrB, clpV/tssH, cpsACP, dotU/tssL, entA, entB, entC, entE, entF, fepA, fepB, fepC, fepD, fepG, fes, fimA, fimB, fimC, fimD, fimE, fimF, fimG, fimH, fimI, fimK, galF, gnd, hcp/tssD, impA/tssA, iroE, mrkA, mrkB, mrkC, mrkD, mrkF, mrkH, mrkI, mrkJ, rcsA, rcsB, sciN/tssJ, tssF, tssG, ugd, vasE/tssK, vgrG/tssI, vipA/tssB, vipB/tssC, wzi, ybdA |
